# Optimized Non-Cooperative Spectrum Sensing Algorithm in Cognitive Wireless Sensor Networks

**DOI:** 10.3390/s19092174

**Published:** 2019-05-10

**Authors:** Yangyi Chen, Shaojing Su, Huiwen Yin, Xiaojun Guo, Zhen Zuo, Junyu Wei, Liyin Zhang

**Affiliations:** 1 College of Intelligent Science and Technology, National University of Defense Technology, Changsha 410073, China; kd_chenyy@163.com (Y.C.); susj-5@163.com (S.S.); yinhuiwen18@163.com (H.Y.); z.zuo@nudt.edu.cn (Z.Z.); 2 College of electric and information engineering, Hunan University of Technology, Zhuzhou 411201, China; davidjunyu123@163.com; 3 Logistics department, Unit 93010 of the people’s liberation army, Shenyang 110000, China; kd_paper@163.com

**Keywords:** cognitive wireless sensor networks, spectrum sensing, singular spectrum entropy, multi-resolution analysis

## Abstract

The cognitive wireless sensor network (CWSN) is an important development direction of wireless sensor networks (WSNs), and spectrum sensing technology is an essential prerequisite for CWSN to achieve spectrum sharing. However, the existing non-cooperative narrowband spectrum sensing technology has difficulty meeting the application requirements of CWSN at present. In this paper, we present a non-cooperative spectrum sensing algorithm for CWSN, which combines the multi-resolution technique, phase space reconstruction method, and singular spectrum entropy method to sense the spectrum of narrowband wireless signals. Simulation results validate that this algorithm can greatly improve the detection probability at a low signal-to-noise ratio (SNR) (from −19dB to −12dB), and the detector can quickly achieve the best detection performance as the SNR increases. This algorithm could promote the development of CWSN and the application of WSNs.

## 1. Introduction

The emergence of cognitive wireless sensor networks (CWSN) has significantly extended the applications of wireless sensor networks (WSNs) due to their ability to use a dynamic spectrum to increase the available spectrum resources. A variety of spectrum sharing technology has been applied to the study of dynamic spectrum utilization, enabling spectrum resource management to change from static to dynamic. Spectrum sensing technology is the basis of spectrum sharing, which can acquire spectrum use information in wireless networks by using signal detection and processing methods. In other words, it can dynamically detect the change of the communication environment and obtain an available communication channel in time.

Recent work has shown that cooperative spectrum sensing can effectively improve the sensing performance of CWSN. However, cooperative spectrum sensing increases the communication load and computing load of sensor nodes and reduces the life-time of nodes and the network. We have previously studied various types of cooperative spectrum sensing architectures and algorithms; although the requirement of node computing power has been reduced, the problem of the high requirement of communication ability has still not been solved. Moreover, kinds of non-cooperative spectrum sensing algorithms have been studied, but the detection performance cannot meet the requirements of wireless sensor networks.

Here, we present a novel non-cooperative spectrum sensing algorithm in which the spectrum can be sensed by using the multi-resolution technique, phase space reconstruction method, and singular spectrum entropy method. Distinct from previous reports, the singular spectrum entropy method is applied to the non-cooperative spectrum sensing algorithm, which makes use of the information entropy theory for spectrum sensing without any prior knowledge of signals, and the detection performance can be greatly improved at a low signal-to-noise ratio (SNR). The main contributions of this paper are as follows:

1. We present a CWSN architecture based on non-cooperative spectrum sensing, which reduces the computing requirements for nodes and the communication capability of CWSN.
2. We propose a spectrum sensing algorithm based on multi-resolution singular spectrum entropy, which can enhance the stability and reliability of communication links between CWSN nodes.
3. The proposed algorithm is verified by MATLAB, and the simulation results show that the detection performance of the algorithm is significantly improved compared with the previous algorithm.

The remainder of the paper is organized as follows. In Section 2, related works are reviewed. The CWSN architecture and system model are introduced in Section 3. In Section 4, we propose a detection algorithm based on multi-resolution singular spectrum entropy (MRSSE algorithm). Section 5 discusses the detection threshold of the algorithm. Section 6 presents the obtained simulation results. Finally, in the last section, we discuss the results and draw conclusions.

## 2. Related Works

With the continuous increase of wireless services, traditional WSNs operating over unlicensed spectra are facing unprecedented challenges to guarantee network performance. As a promising solution for the spectrum scarcity of WSNs, CWSN has been shown to improve network performances. The authors of [1,2,3] reviewed of cognitive wireless sensor networks is presented, and these papers introduce the concept, architecture and problems to be addressed for cognitive wireless sensor networks. The authors of [4] conducted a survey on spectrum resource allocation schemes for cognitive radio sensor networks (CRSN) which concentrate on the classification and performance index of schemes. The authors of [5] investigated the optimal sensing policy and access policy for energy harvesting cognitive radio sensing networks. A novel resource allocation solution with which the detected available time of the channels can be maximized is proposed for heterogeneous cognitive radio sensing networks (HCRSNs) in Reference [6]. The authors of [7] proposed an energy-aware mode switching strategy and cluster head selection algorithm for the better energy management of CRSN for IoT. The authors of [8] determined the conditions of sensing and accessing licensed channels for potential energy consumption reduction. Based on this, two sequential channel sensing and accessing schemes are proposed for intra- and inter-cluster data transmission which can achieve optimal energy efficiency in clustered CRSN [9]. The authors of [10] highlighted MAC protocols, but not MAC layers for CRSN.

Beyond this, we can conclude that the main research content of CWSN is how to use cognitive technology rather than sensing technology. However, the performance of sensing technology also affects WSN energy consumption, and sensing technology is mainly studied in cognitive radio. Now the research into spectrum sensing technology can be divided into non-cooperative spectrum sensing and cooperative spectrum sensing. The non-cooperative spectrum sensing technology is the main spectrum sensing technology in CWSN which can be summarized as follows [11,12]: energy detectors [13,14,15,16], feature detection [17,18,19,20], and matched filter detection [21,22,23]. The approaches are summarized in Table 1, which shows the algorithm, its limitation and the relevant recent references [24]. 

We can determine from Table 1 that matched filter detection and feature detection require prior knowledge of the primary network [25,26,27], but there is often a lack of prior knowledge of the primary network in CWSN. Therefore, these methods are difficult to apply to CWSN, which is based on non-cooperative spectrum sensing. Considering that an energy detector needs to overcome the influence of noise energy on the detection performance [28,29], it is difficult to use in low SNR environments, although a series of algorithms have been proposed at present.

## 3. System Model

### 3.1. CWSN Architecture

**Figure 1:**
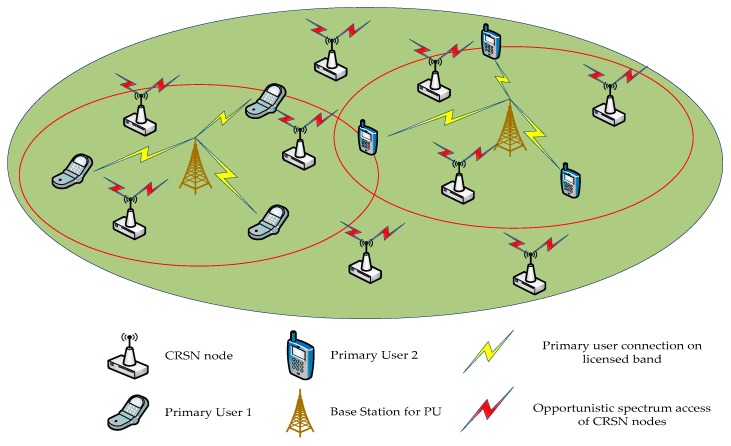
The structure of cognitive wireless sensor networks. CRSN: cognitive radio sensor network.

In cognitive radio, cooperative spectrum sensing is generally applied to obtain higher detection performance. In cooperative sensing, the data collected by the sensing node need to be transmitted to the central node for spectrum sensing. However, this is difficult to implement in CWSN. Firstly, it is hard to realize data transmission between CWSN nodes through an independent channel due to the shortage of spectrum resources. Secondly, it is unwise to add additional communication links between CWSN nodes, especially in the case of limited energy. Finally, in the topology of CWSN, the center node is variable and energy-intensive, which causes the energy consumption of sensor nodes to rise and reduces the life-time of the network. According to the analysis, it can be seen that the cooperative sensing model is not suitable for cognitive wireless sensor networks, while the non-cooperative sensing model can be used. Based on these, this paper proposes the CWSN architecture, which is shown in Figure 1.

On the basis of Figure 1, we assume that CWSN nodes do not have any prior knowledge of their external electromagnetic environment, and each node is a single antenna structure in our research in order to be more realistic, thus, only one antenna can transmit data. In addition, only one channel can be detected per time slot. 

For the convenience of study, we also make the following assumptions: the primary user signal (PU) and noise signal are independent from each other, the PU signals are also independent from each other, and the noise signal is an independent and identically distributed Gaussian white-noise signal.

### 3.2. Binary Hypothesis Model

In this paper, binary hypothesis is used to analyze spectrum sensing [12]. Let y(t) be the continuous-time received signal and $T_{s}=1/f_{s}$ be the sampling period; thus, the received signal samples of sensing node is:

(1) $$y[n]=y(nT_{s}).$$

There are two hypotheses for the received signal: $H_{0}$, meaning that only the noise (no PU signal) exists; and $H_{1}$, meaning that both the PU signal and the noise exist. The received signal samples under the two hypotheses are given, respectively, as follows:

(2) $$\begin{array}{l} H_{0}:y[n]=w[n]\\ H_{1}:y[n]=x[n]+w[n] \end{array},$$

where x[n] is the PU signal sample with an unknown channel with unknown signal distribution, w[n] is the additive white Gaussian noise (AWGN), and each sample of w[n] is assumed to be independent identical distribution, with zero mean and $\sigma_{w}^{2}$ variance (unknown), n=1,2,…,N−1, and N indicates the sample size.

Spectrum sensing is a signal detecting problem on the basis of statistical decision theory. There are two important probabilities: the probability of detection ($P_{d}$) and probability of false alarm ($P_{f}$). $P_{d}$ is the probability of the algorithm correctly detecting the presence of a primary signal under hypothesis $H_{1}$. $P_{f}$ is the probability of the algorithm falsely declaring the presence of a primary signal. The basic principle of spectrum dynamic access is that the unlicensed operator cannot interfere with the licensed users’ work; secondary users are required to abandon the channel when there is a primary user in the channel. Thus, the aim of spectrum sensing is to ensure the best $P_{d}$ under the constraint condition of $P_{f}$. 

### 3.3. Performance Index

In this research, we select two indexes which are mostly used in NP (Neyman–Pearson) detectors as the performance indexes of the spectrum sensing algorithm: the detection probability ($P_{d}$) curve and ROC (receiver operating characteristic). The detection probability curve reflects the detection capability of the spectrum sensing algorithm directly, and the ROC is a coordinate graph analysis tool which is used to select the best detection model and set the model threshold in signal detection theory. We can not only get the optimal $P_{f}$ from ROC, but also compare the performance of two algorithms. Therefore, we can use the detection probability curve and ROC to compare the detection performance of different algorithms to verify the advantages and disadvantages of the algorithm proposed in this paper. The purpose of our study is to obtain the highest detection probability under the given detection conditions, which include SNR, $P_{f}$, signal parameters and so on. We can get the optimal detection condition from the detection probability curve and ROC.

## 4. Detection Based on Multi-Resolution Singular Spectrum Entropy

### 4.1. Algorithm Fundamentals

In this section, we study the wavelet packet decomposition and singular spectrum entropy, which are the basic theory and technology of the MRSSE algorithm proposed in this paper. Wavelet packet decomposition is a typical multi-resolution analysis method which can select the resolution based on the application requirement, and the singular spectrum is an important concept in information theory which can analyze signals accurately without prior knowledge of the signal.

#### 4.1.1. Wavelet Packet Decomposition

**Figure 2:**
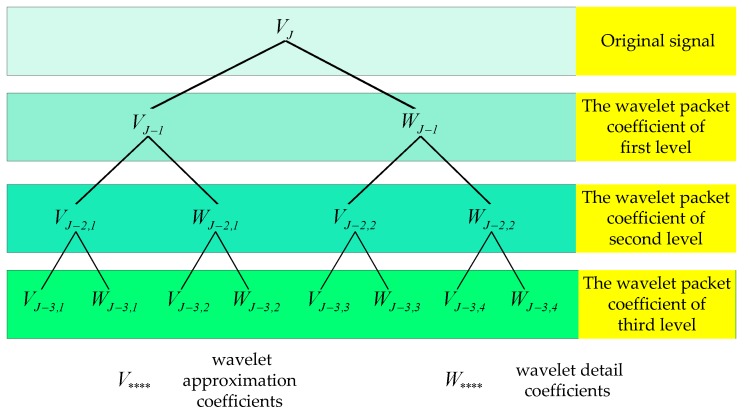
Decomposition process of wavelet packet decomposition (three levels as an example).

Wavelet packet decomposition is an extension of wavelet decomposition. It is a time-frequency analysis method which is more detailed than wavelet analysis, and it decomposes not only the scale space but also the wavelet space of the signal. The process of wavelet packet decomposition is shown in Figure 2.

In Figure 2, $V_{J-2,2}$ is the approximation of $W_{J-1}$, and $W_{J-2,2}$ is the detailed version of $W_{J-1}$; similarly, $V_{J-3,4}$ is the detailed version of $W_{J-2,2}$ and it is the result of $W_{J-1}$ after two-level wavelet packet decomposition. The signal can be divided into 26 different types of decomposition through the three-level wavelet packet decomposition, and Equation (3) describes the most typical decomposition mode. Thus, we can flexibly select the decomposition mode of the signal according to the signal characteristics and analysis requirements.

(3) $$V_{J}=V_{J-3,1}⊕W_{J-3,1}⊕V_{J-3,2}⊕W_{J-3,2}⊕V_{J-3,3}⊕W_{J-3,3}⊕V_{J-3,4}⊕W_{J-3,4}$$

**Figure 3:**
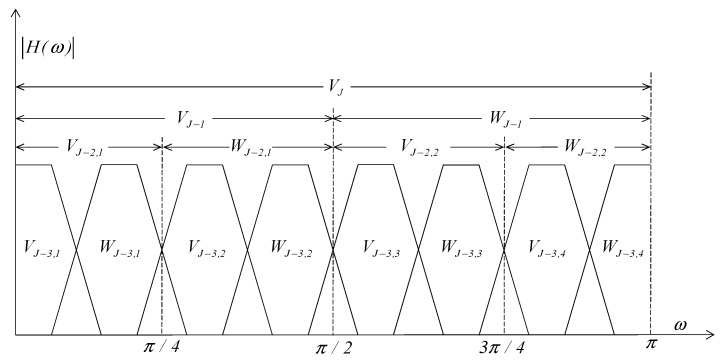
The spectrum characteristics of wavelet packet decomposition.

The spectrum characteristics of wavelet packet decomposition described by Equation (3) are shown in Figure 3. We can see from Figure 3 that the higher the wavelet decomposition level, the finer the resolution of frequency will be. For example, when the decomposition level is two, the frequency resolution is π/4 and when the decomposition level is three, the frequency resolution is π/8.

The result of the wavelet packet decomposition is a series of wavelet packet coefficients: $S_{J,i}$, where *J* is the level of wavelet packet decomposition, and $i=1,2,\ldots ,2^{J}$ is the number of *J* level wavelet coefficients. $S_{J,i}$ represents the feature of the signal in the frequency band, thus, we can analyze the wavelet packet coefficients to get the feature of the signal. In Figure 2 and Figure 3, the wavelet packet coefficient $S_{J,i}$ is relative to the decomposed coefficient ($V_{****}$ or $W_{****}$) respectively, for example, $S_{3,5}=V_{J-3,3}$.

#### 4.1.2. Singular Spectrum Entropy

Singular spectrum entropy is an analysis method for array signals, however, in this study, the wavelet packet coefficient is a sequence signal. Thus, when using singular spectrum entropy for spectrum sensing, the wavelet packet coefficient needs to be preprocessed to meet the requirements of the singular spectrum entropy analysis method. Based on this, we constructed the trajectories matrix in the phase space. We suppose that the dimension of the trajectories matrix is *m*, the delay time is *L*, and the length of row vector of matrix is $N_{m}$ [30]. Thus, we can get the correlation of these parameters as follows:

(4) $$N_{m}=N-(m-1)L.$$

We can get the trajectories matrix of wavelet packet coefficients in a phase space with Teken’s embedding theorem [30]:

(5) $$Y=\left[\begin{array}{cccc} y[1] & y[2] & \cdots & y[N_{m}]\\ y[1+L] & y[2+L] & \cdots & y[N_{m}+L]\\ \vdots & \vdots & \vdots & \vdots \\ y[1+\left(m-1\right)L] & y[2+\left(m-1\right)L] & \cdots & y[N_{m}+\left(m-1\right)L] \end{array}\right].$$

Here, we transform the time sequence into a signal sample matrix. The covariance matrix of **Y** can be calculated by Equation (6).

(6) $$C_{x}=YY^{H}$$

The singular value decomposition of the covariance matrix can be written as follows:

(7) $$C_{x}=U^{T}\Lambda U,$$

where U is the orthogonal matrix of eigenvectors, and Λ is the diagonal matrix composed by singular values, also called the singular spectrum. Thus, $\Lambda =\mathrm{diag}\{\lambda_{1},\lambda_{2},\ldots ,\lambda_{m}\}$, $\lambda_{i}$ is the singular value of $C_{x}$, and the singular value is ordered: $\lambda_{1}\geq \lambda_{2}\geq \ldots \geq \lambda_{m}\geq 0$. Based on information theory, the definition of singular spectrum entropy is [31,32]:

(8) $$h=-\sum_{i=1}^{m}p_{i}\log p_{i},$$

where $p_{i}$ represents the contribution rate of each singular value to the whole singular spectrum. The $p_{i}$ can be calculated by:

(9) $$p_{i}=\lambda_{i}/\sum_{i=1}^{m}\lambda_{i}.$$

The singular spectral entropy of the signal can effectively represent the average uncertainty of the signal, and the feature of the signal under two hypotheses can be extracted.

### 4.2. Proposed Algorithm

Multi-resolution analysis can help to fully understand the characteristics of signals at different resolutions by analyzing signals at different scales and resolutions. Singular spectrum entropy is a kind of information entropy, and its value reflects the signal characteristics. The lower the singular spectrum entropy, the more ordered the signal. In spectrum sensing, the randomness of the signal is related to the state of the signal. Under hypothesis $H_{1}$, the signal is more ordered than the signal under hypothesis $H_{0}$. Based on the above advantages of multi-resolution analysis and singular spectrum entropy, this paper proposes a non-cooperative spectrum sensing algorithm based on multi-resolution singular spectrum entropy, which is the MRSSE algorithm. 

It can be seen from Equation (2) that only noise signals are received by sensor nodes under hypothesis $H_{0}$, and the noise signal is white Gaussian noise with zero mean and $\sigma_{w}{}^{2}$ variance. Thus, wavelet packet coefficients, namely the results of wavelet packet decomposition, can be regarded as a series of band-limited white noise, $S_{J,i}[n]=w_{i}[n]$, and we can get the covariance matrix of the wavelet packet coefficient trajectories matrix by Equations (5) and (6). The autocorrelation coefficient of white noise can be described by Equation (10):

(10) $$R_{n}(\tau )=\sigma_{w}{}^{2}\frac{N_{0}}{2}\delta (\tau ).$$

The covariance matrix can be written as:

(11) Cx=σw2[N020⋯00N02⋯0⋮⋮⋱000⋯N02].

So, the singular value of $C_{x}$ is:

(12) λ1′=λ2′=…=λm′=σw2N02.

According to the definition of singular the spectrum computed by Equation (9), we obtain:

(13) p1′=p2′=…=pm′=1M.

So, we can calculate the singular spectrum entropy by using $p_{i}{}^{\prime }$ to substitute $p_{i}$ in Equation (8):

(14) $$\begin{array}{c} h_{i}=-\sum_{i=1}^{m}\frac{1}{m}\log\frac{1}{m}\\ =-\sum_{i=1}^{m}\frac{1}{m}(\log1-\log m),\\ =\sum_{i=1}^{m}\frac{1}{m}\log m=\log m \end{array}$$

(15) $$D=\sum_{i=1}^{2^{J}-1}d_{i}^{2}=\sum_{i=1}^{2^{J}-1}{\left(h_{i+1}-h_{i}\right)}^{2}=\sum_{i=1}^{2^{J}-1}{\left(\log m-\log m\right)}^{2}=0.$$

Therefore, in the $H_{0}$ hypothetical case, the singular spectral entropy of each wavelet packet coefficient is equal, and the sum of squares of the distance between adjacent singular spectral entropy is written as Equation (15). 

According to the definition of information theory, in the $H_{0}$ hypothetical case, the received signal contains only a noise signal, the system state is stable and orderly, and the entropy value of each wavelet packet coefficient is minimal and equal. In the $H_{1}$ hypothetical case, with the addition of the PU signal, the stability of the system is reduced, and so the singular spectral entropy of some wavelet packet coefficients will increase. Thus, there is a big difference between the two hypotheses in the sum of squared distances of adjacent singular spectral entropy, and we set γ as our decision criterion:

(16) $$\left\{ \begin{array}{l} D>\gamma \to H_{1}\\ D<\gamma \to H_{0} \end{array}\right..$$

**Figure 4:**
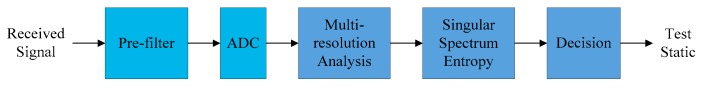
Block diagram of the non-cooperative spectrum sensing algorithm based on multi-resolution singular spectrum entropy.

Based on the above analysis, the MRSSE algorithm which we proposed in this paper is summarized as Algorithm 1. The structure of Algorithm 1 is shown in Figure 4.
**Algorithm 1** Spectrum sensing algorithm with multi-resolution singular spectral entropy **Input:** Network parameters, decision threshold ($P_{f}$) and sampled data y[n]**Output:** The state of the channel perceived by the spectrum sensing algorithm (Cs)1:Initialization, parameter setting: γ,m,L,J
2:**for all**y[n]∈R**do**3:   calculate wavelet packet coefficients: $S_{J,i}$, *i* = 1, 2, …, 2*^J^*4:   **for all**
$i\leq 2^{J}$
**do**5:      calculate the singular spectral entropy: $h_{i}$
6:   **end for**7:   **for all**
$2\leq i\leq 2^{J}$
**do**8:      $d_{i}=h_{i}-h_{i-1}$9:   **end for**10:   $D=\sum_{i=2}^{2^{J}}d_{i}$11:   **if**
D≥γ
**then**12:      return: $Cs\leftarrow H_{1}$
13:   **Else**14:      return: $Cs\leftarrow H_{0}$
15:   **end if**16:**end for**

**Figure 5:**
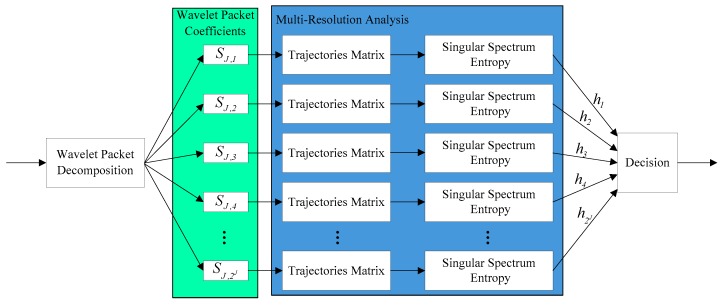
Signal processing flow in the multi-resolution analysis module, singular spectrum module entropy and decision module.

In the structure shown in Figure 4, the wireless signal is converted to a digital signal through the ADC module, and the multi-resolution analysis module, singular spectrum entropy module and decision module are the core parts of the algorithm. The processing flow of the signal in these modules is shown in Figure 5.

The computational complexity of MRSSE is higher than energy detection algorithm. In this paper, we focus on the detection performance of the algorithm, and so the computational complexity of the algorithm is not optimized. Traditionally, the computational complexity of eigenvalue decomposition is related to the size of the matrix (*n*), generally O(n^3). However, in the MRSSE algorithm, the matrix size of eigenvalue decomposition is fixed (*L*), therefore, the time complexity of eigenvalue decomposition is O(1). The complexity of wavelet packet decomposition is mainly related to the signal length (*N*) and the number of decomposition layers (*J*), the computational complexity is higher than O(MlogM), and the larger the signal size and the more levels of decomposition, the higher the computational complexity of the algorithm.

## 5. Detection Threshold

We can determine that the selection of detection threshold (γ) is independent of the variance ($\sigma_{w}^{2}$) of the noise signal from Equations (12) and (13), thus, the MRSSE algorithm proposed in this paper is insensitive to the power of noise signal. Considering the uncertainty of noise, we make the following assumption.

**Assumption** **1.** *in the $H_{0}$ hypothetical case, the singular spectrum entropy ($h_{i}$) of each wavelet packet coefficient is an independent identical distribution (i.i.d.), and it obeys normal distribution with logm mean and $\sigma_{s}^{2}$ variance; the probability density function is:*

(17) $$f(h_{i})=\frac{1}{\sqrt{2\pi }\sigma_{s}}\exp(-\frac{{\left(h_{i}-\log m\right)}^{2}}{2\sigma_{s}{}^{2}}).$$

The distance between adjacent singular spectrum entropy is defined as $d_{i}$:

(18) $$d_{i}=h_{i+1}-h_{i}.$$

So, $d_{i}∼CN(0,\sigma_{s}^{2})$ and the probability density function of $d_{i}$ can be written as:

(19) $$f(d_{i})=\frac{1}{\sqrt{2\pi }\sigma_{s}}\exp(-\frac{d_{i}{}^{2}}{2\sigma_{s}{}^{2}}).$$

Normalize $d_{i}$:

(20) $$d_{i}{}^{\prime }=d_{i}/\sigma_{s}.$$

So, $d_{i}{}^{\prime }$ is a standard normal variable; according to Equation (15), we can obtain $D^{\prime }$ which is normalized.

(21) $$D^{\prime }=\sum_{i=1}^{2^{J}-1}{\left(d_{i}{}^{\prime }\right)}^{2}=\sum_{i=1}^{2^{J}-1}{\left(d_{i}/\sigma_{s}\right)}^{2}=\sum_{i=1}^{2^{J}-1}\frac{{\left(h_{i+1}-h_{i}\right)}^{2}}{\sigma_{s}{}^{2}}.$$

The variable $D^{\prime }$ follows a chi-squared distribution with $2^{J}-1$ freedom, so the probability density function and cumulative distribution function are described as follows:

(22) f(D′;k)={x(k2−1)e−x22k2Γ(k2),x>0;0,otherwise

(23) Q(D′;k)=γ(k2,x2)Γ(k2),

where *k* is the freedom of chi-squared distribution. Based on the definition of $p_{f}$, we obtain:

(24) $$P_{f}=P(D^{\prime }>\gamma |H_{0})=\int_{\gamma }^{\infty }f(t)dt.$$

So, the threshold can be determined by Equation (25):

(25) $$\lambda =D^{\prime }+Q^{-1}(1-P_{f}),$$

where $D^{\prime }=0$, and the determination of the threshold value is only related to the false alarm rate. Table 2 captures part of the probability distribution table of chi-squared distribution.

## 6. Simulation Results 

In order to verify the feasibility of the MRSSE algorithm, MATLAB is used for simulation. In this paper, we designed a simulation environment with fixed and variable parameters. The fixed parameters are the primary user’s signal parameters and the parameters that do not affect the performance of the algorithm, mainly including the signal modulation format (FDM), sampling length of the signal (*N* = 100,000), sampling frequency of the signal (*fs* = 10,000), and wavelet function (db6). The variable parameters are the parameters that affect the performance of the algorithm, mainly including the signal-to-noise ratio of received signal (SNR), standard deviation of the noise signal ($\sigma_{w}{}^{2}$), wavelet decomposition layer number (*J*), phase space trajectories matrix dimensions (*m*), phase space reconstruction signal delay time (*L*), and false alarm rate ($p_{f}$).

**Figure 6:**
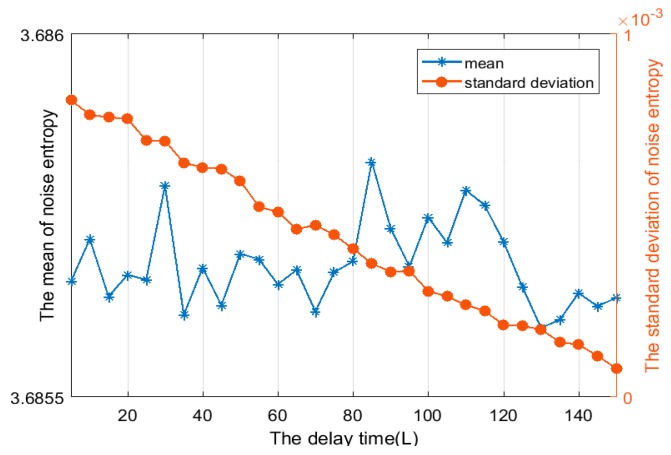
The relationship between distribution characteristics and the delay time of the trajectories matrix (*L*) under hypothesis $H_{0}$ (*m* = 40, *J* = 4). The mean of the singular spectrum entropy is approximately equal to the logm=log40=3.688, and the standard deviation of singular spectrum entropy is similar to zero.

**Figure 7:**
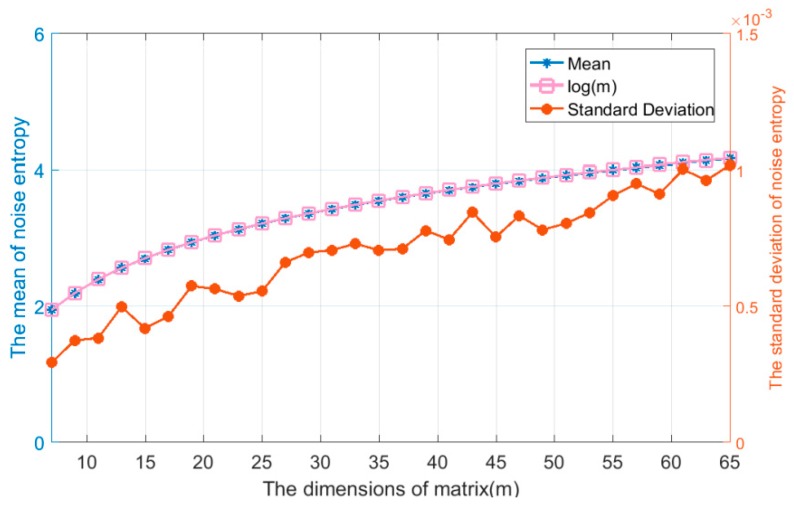
The relationship between the distribution characteristics and dimension of the trajectories matrix (*m*) under hypothesis $H_{0}$ (*L* = 30, *J* = 4). The mean of the singular spectrum entropy is approximately equal to the logm curve, and the standard deviation of singular spectrum entropy is similar to zero.

Firstly, we test Assumption 1 which was proposed in the study of the algorithm threshold setting. We set up multiple sets of simulation conditions, and the results are shown in Figure 6 and Figure 7. The results show that due to the uncertainty of noise, the distribution of singular spectrum entropy satisfies the normal distribution with mean logm and variance $\sigma_{s}^{2}$. 

**Figure 8:**
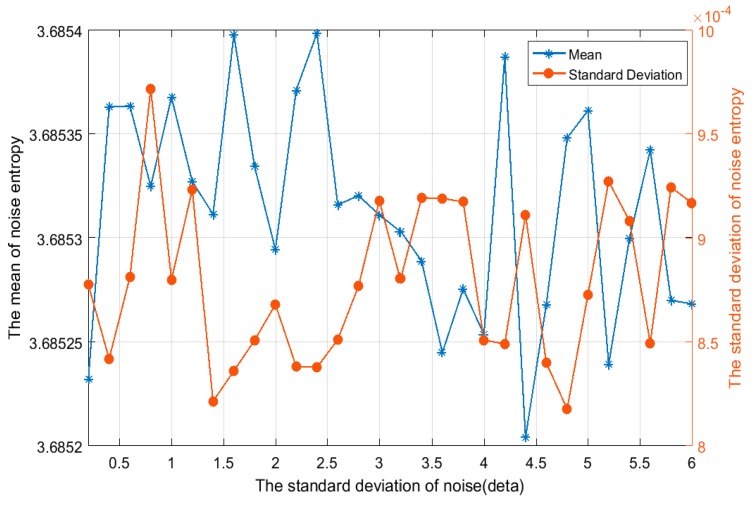
The relationship between distribution characteristics and standard deviation of the noise signal ($\sigma_{w}{}^{2}$) under hypothesis $H_{0}$ (*m* = 40, *L* = 20, *J* = 4). The mean of the singular spectrum entropy is approximately equal to the logm=log40=3.688, and the standard deviation of singular spectrum entropy is similar to zero.

Then, the relationship between the distribution of signal singular spectrum entropy and the energy of the noise signal is further verified. As shown in Figure 8, under hypothesis $H_{0}$, the distribution of the signal singular spectrum entropy is independent of the energy of noise signal. This conforms to the theoretical analysis shown in Equations (13) and (14).

**Figure 9:**
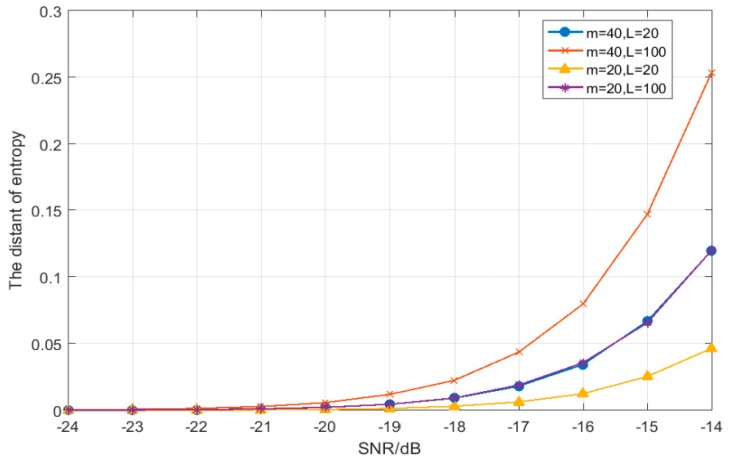
The relationship between SNR and the sum of the squares of the distance of adjacent singular spectrum entropy under hypothesis $H_{1}$ (*J* = 4).

Finally, we study the sum of the distances’ square of adjacent singular spectrum entropy under hypothesis $H_{1}$, and the results are shown in Figure 9. As we can see from Figure 9, when the SNR is very low, the difference between the multi-resolution singular spectrum entropies is very small, and the sum of the distances’ square of adjacent singular spectrum entropy is basically zero. As the SNR increases, the difference between the values of the multi-resolution singular spectrum entropy increases, and the sum of squares of the distances also increases. In addition, the sum of the distances’ squares of adjacent singular spectrum entropy is also related to the simulation parameters. According to Figure 9, when *m* = 40 and *L* = 100, the sum of the distances’ squares is the largest. 

**Figure 10:**
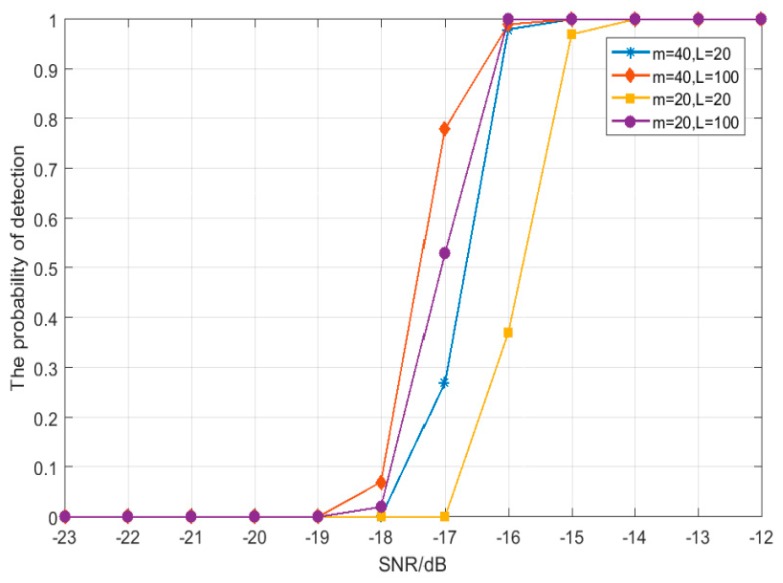
The relation between detection probability and SNR with different *m* and *L* combinations and a fixed *J* (*J* = 4).

**Figure 11:**
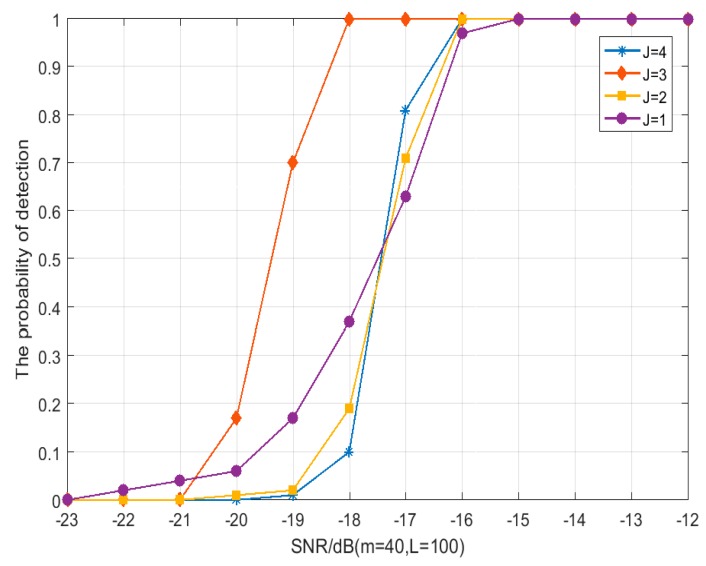
The relation between detection probability and SNR with different *J* and fixed m and *L* (*m* = 40, *L* = 100).

Based on this, the simulation parameters with the best detector performance are studied by adjusting the variable parameters, and the simulation results are shown in Figure 10 and Figure 11, thus, we get the best parameter combination in this paper: *J* = 3, *m* = 40, and *L* = 100.

**Figure 12:**
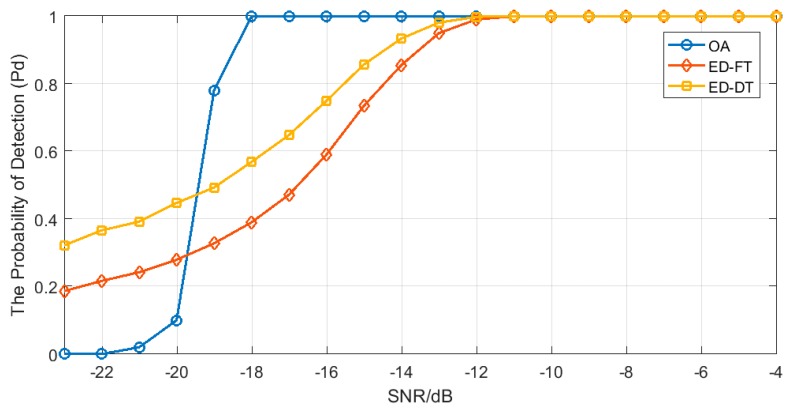
Comparison of the detection probability under different SNR between the multi-resolution singular spectrum entropy (MRSSE) algorithm and traditional spectrum sensing algorithms (OA: MRSSE algorithm; ED-FT: the energy detection algorithm with fixed threshold; ED-DT: the energy detection algorithm with adaptive threshold).

**Figure 13:**
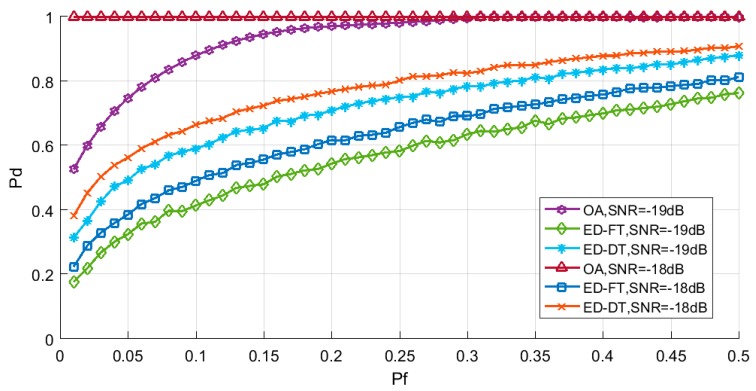
Comparison of the receiver operating characteristic (ROC) curve between the MRSSE algorithm and traditional spectrum sensing algorithms (OA: MRSSE algorithm; ED-FT: the energy detection algorithm with fixed threshold; ED-DT: the energy detection algorithm with adaptive threshold) under different SNRs. *Pd* is probability of detection and *Pf* is probability of false alarm.

After obtaining the optimal performance parameters for the detector, we compared the MRSSE algorithm with the most widely used non-cooperative spectrum sensing algorithm in cognitive wireless sensor networks (the energy detection algorithm with a fixed threshold and the energy detection algorithm with ab adaptive threshold), and the simulation results are shown in Figure 12 and Figure 13. It can be seen from the results that the detection performance of the MRSSE algorithm is better than the other two algorithms.

## 7. Discussion and Conclusions

We have shown that the spectrum sensing algorithm which is based on multi-resolution singular spectral entropy can greatly improve detection performance, especially at low SNR, and the algorithm is both sensitive and reliable when we study spectrum sensing based on a non-cooperative design.

Detection performance was examined by comparing the detection probability curve of the MRSSE algorithm and the conventional algorithms ([15,29]), with the comparison revealing a significant performance increase. In comparison to previous studies, the MRSSE algorithm exhibits two principal advantages: firstly, in the case of low SNR (from −19.5dB to −12dB), the detection probability of the algorithm is significantly higher than that of the traditional algorithms; secondly, the ROC curve is steeper, and the detector can quickly achieve the best detection performance. We also found in the study that the detection probability of the MRSSE algorithm is lower than the traditional algorithms when the SNR is lower than −19.5dB. This is because the energy of the primary user signal is relatively low in the case of low SNR (lower than −19.5dB), and the energy of the primary user signal is lower in wavelet packet coefficients after wavelet packet decomposition, therefore, the detector may regard the primary user signal as a noise signal.

However, spectrum sensing is divided into the wideband spectrum sensing problem and narrowband spectrum sensing problem in CWSN. In this paper, the MRSSE algorithm is designed to improve the performance of narrowband spectrum sensing while wideband spectrum sensing is the trend of spectrum sensing technology development. Hence, in our following research, we will mainly focus on the study of wideband spectrum sensing technology.

The MRSSE algorithm we proposed in this paper shows significantly better detection performance than conventional algorithms in the case of low SNR. Additionally, it is a promising method for solving the problem of non-cooperative narrowband spectrum sensing.

## Figures and Tables

**Table 1:** Summary for various non-cooperative sensing techniques for the cognitive wireless sensor network (CWSN). SNR: signal-to-noise ratio.

Sensing Technique	Advantages	Limitations	Relevant Recent References
Energy Detector	● Does not require prior knowledge about the primary network ● Simple to design, implement, and has less complexity	● Cannot discriminate between primary signal and noise ● Cannot perform well for low SNR ● Vulnerable to noise uncertainty	[13,14,15,16,28,29]
Feature Detection	● Sensing performance is highly reliable, can detect signals with low SNR ● Robust to noise uncertainty	● Prior knowledge of the primary network ● Higher accuracy requires a longer length of known sequences that results in lower efficiency of the spectrum ● Slower sensing compared to energy detection	[17,18,19,20,26,27]
Matched Filter	● Optimal sensing performance, maximizes the received SNR ● Less time needed to achieve high processing gain	● Prior knowledge of the primary network ● Computational complexity depends on the primary network ● Dedicated sensing receiver required for synchronization at each SU	[21,22,23,25]

**Table 2:** Probability distribution table of chi-squared distribution.

K	$P(p_{f})$							
	0.995	0.95	0.9	0.85	0.15	0.1	0.05	0.005
1	…	…	0.02	0.04	2.07	2.71	3.84	7.88
3	0.07	0.35	0.58	0.80	5.32	6.25	7.81	12.84
7	0.99	2.17	2.83	3.36	10.75	12.02	14.07	20.28
15	4.60	7.26	8.55	7.90	20.60	22.31	25.00	32.80
